# Impact of timing HAART initiation on immune status and clinical course in the cohort of the German competence network for HIV/AIDS

**DOI:** 10.1186/1758-2652-13-S4-P16

**Published:** 2010-11-08

**Authors:** A Plettenberg, NH Brockmeyer, B Haastert, S Dupke, CK Schewe, M Rausch, M Hower, G Arendt, K Jansen, HIV KompNet

**Affiliations:** 1 Ifi-Institut, Hamburg, Germany; 2 St. Josef-Clinic, Department of Dermatology, Bochum, Germany; 3 mediStatistica, Neuenrade, Germany;; 4 Clinical practice Driesener Strasse, Berlin, Germany; 5 ICH Study Centre, Hamburg, Germany; 6 Clinical Practice Fuggerstrasse, Berlin, Germany; 7 City clinic, Dortmungd, Germany; 8 University Clinic, Department of Neurology, Dusseldorf, Germany; 9 Competence Network for HIV/AIDS, Department of Dermatology, St. Josef-Hospital, Bochum, Germany

## Purpose of the study

Optimal time of HAART initiation is still unknown. Several studies investigating this issue showed inconsistent results. Our study investigated whether there is a clinical benefit as to treated patients (pts) by an earlier start of therapy at CD4 between 350-449c/µl, compared to the range of 250-349c/µl. An analysis was conducted on basis of the open, retrospective and prospective, multi-center and nationwide cohort of the Competence Network for HIV/AIDS.

## Methods

For analysis, pts had to be observed at least 3 months before initiation of HAART. Medication had to start later than 1996, with at least three substances. At time of therapy start (t_o_), pts had to have 250c/µl <CD4-cells<450c/µl, no prior AIDS defining conditions and CD4 cells never below 200c/µl. Afterwards, pts were stratified in groups by initial CD4 cells between 250-349c/µl (group 1) and 350-449c/µl (group 2). Primary outcomes death, AIDS and first drop of CD4 cell count/µl<200 cells were evaluated as censored event times between t_o_ and the date of first event resp. last observation. Time dependent probabilities of event free intervals since start of HAART were estimated by Kaplan-Meier estimation, compared by Log-rank-tests. Cox-regression models were fitted, adjusted for time since infection.

## Summary of results

822 pts met inclusion criteria. Group 1 consisted of 526 pts, group 2 of 296. Mean observation time in group 1 was 5.1 years/pt (2,683 years overall), in group 2 4.9 years/pt (1,450 y overall). In group 1, 0.64 deaths occurred per 100 pt years vs. 0.17 events in group 2. 1.38 AIDS events were developed per 100 pt years vs. 0.78 events in group 2. In group 1, 2.64 per 100 pts years dropped <200c/µl vs. 0.77 in group 2. Kaplan-Meier estimations showed borderline significant difference as to developing death (ten years probabilities for having no event as to death: group1 94%, group2: 97%, p=0.063), significant difference as to CD4 drop<200c/µl (group1 80%, group2: 94%, p=0.0004), but no significant differences as to AIDS (group1 92%, group2: 90%, p=0.219). Hazard ratio (group 2 vs. 1) was significant for CD4 drop<200c/µl (0.302, p=0.001), but not for death (0.268, p=0.0829) and AIDS (0.577, p=0.153).

## Conclusions

Results showed a tendency for better outcome of pts by start of therapy with CD4 cell count ≥350c/µl regarding death, clear evidence regarding first CD4 drop <200. These results may be a hint to start therapy earlier.

